# Cutaneous microvascular occlusion syndrome as the first manifestation of catastrophic lupus-associated antiphospholipid antibody syndrome: a case report

**DOI:** 10.1186/s13256-023-04068-9

**Published:** 2023-08-22

**Authors:** Nastaran-Sadat Hosseini, Sharareh Babaei, Hamid Rahimi, Alaleh Gheissari, Banafsheh Sedaghat, Mahsa Pourmahdi-Boroujeni, Bahareh Abtahi-Naeini

**Affiliations:** 1 School of Medicine, Isfahan University of Medical Sciences, Isfahan, Iran; 2 Department of Pediatric Intensive Care Unit, Child Growth and Development Research Center, Research Institute for Primordial Prevention of Non-Communicable Disease, Imam Hossein Children’s Hospital, Isfahan University of Medical Sciences, Isfahan, Iran; 3 Department of Pediatric Infectious Diseases, School of Medicine, Pediatric Cardiovascular Research Center, Imam Hossein Children’s Hospital, Isfahan University of Medical Sciences, Isfahan, Iran; 4 Isfahan Kidney Diseases Research Center and Department of Pediatric Nephrology, Isfahan University of Medical Sciences, Isfahan, Iran; 5 Department of Rheumatology, Shariati Hospital, Isfahan, Iran; 6 Student Research Committee, Isfahan University of Medical Sciences, Isfahan, Iran; 7 Pediatric Dermatology Division of Department of Pediatrics, Imam Hossein Children’s Hospital, Isfahan University of Medical Sciences, Isfahan, Iran

**Keywords:** Microvascular occlusion syndrome, Retiform purpura, Antiphospholipid Antibody Syndrome Systemic Lupus Erythematous

## Abstract

**Background:**

Antiphospholipid syndrome (APS), defined by thrombotic events or obstetric complications in the presence of persistently high antiphospholipid antibodies, is characterized by a wide variety of clinical presentations and the effects of vascular occlusion can impact almost any organ system or tissue. Since adult-onset APS classification criteria are not well verified in pediatrics (where pregnancy-related problems are rare), estimating childhood prevalence is challenging. Stroke and pulmonary embolism are thromboembolic events occurring in children that can cause considerable long-term morbidity. Children with APS are more prone to recurrent thromboembolism than adults. Cutaneous symptoms are prominent and typically represent the first clue of APS. Although dermatologic findings are exceedingly heterogeneous, it is essential to consider which dermatological symptoms justify the investigation of antiphospholipid syndrome and the required further management.

**Case presentation:**

We describe a seven-year-old Iranian boy with retiform purpura and acral cutaneous ischemic lesions as the first clinical presentation of antiphospholipid syndrome in the setting of systemic lupus erythematous.

**Conclusion:**

APS in pediatrics, is associated with a variety of neurologic, dermatologic, and hematologic symptoms. Therefore, it is essential for pediatricians to be aware of the rare appearance of Catastrophic APS as an initial indication of APS.

## Background

Antiphospholipid syndrome (APS) is a rare autoimmune condition in children. Pediatric APS appears to be under-recognized because there are no universally accepted and verified criteria. For most researchers, the term "pediatric APS" applies if the classification criteria for adult-onset APS are fulfilled in patients below the age of 18 years [1–3]. Pediatric and adult-onset APS have distinct laboratory and clinical features. Adult-onset disease has a pronounced female predominance, whereas childhood disease has a more even gender split [4].

Catastrophic APS (CAPS) is a serious medical and potentially life-threatening state when occlusions in the microvascular system extend to critical organs such as the heart, lungs, and kidneys. In children, the prognosis for CAPS is poor, with roughly one-fourth of CAPS patients leading to death [1, 2]. 

The occurrence of systemic thromboembolic events is commonly documented in APS patients. One of the peculiar aspects of thrombosis is the presence of isolated microthrombosis. The cutaneous microvascular occlusion syndrome (MVOS) is a prominent form of micro thrombosis that manifests in the skin; It is a group of clinically significant skin lesions characterized by retiform purpura, purpura fulminant, non-inflammatory bland necrosis, and skin ulcers [5].

Rarely, a cutaneous manifestation of APS, including livedo reticularis, necrotizing vasculitis, thrombophlebitis, ulcerations, or pointed subungual hematoma, may be the first presentation of APS. In addition, pediatric APS has been associated with exceptional cases of epidermal necrosis and intravascular thrombosis in the dermal vessels [6].

Vessel wall damage or vessel lumen obstruction (thrombotic or embolic disease) are two possible mechanisms that can compromise natural blood flow—identifying the mechanisms that cause retiform purpura can aid in determining the etiology of this crucial dermatologic sign. Applying a systematic approach promotes the precision and agility of diagnosis and finding an appropriate treatment [5].

It is clear that skin can be potentially involved in the course of APS. In this regard, all should be aware of the simultaneous presentation of cutaneous microthrombi representing as retiform purpura in the setting of pediatric APS. Only a few cases have documented the dermatological manifestation of cutaneous MVOS in pediatric APS with or without systemic lupus erythematosus (SLE), suggesting that the problem is underdiagnosed [7].

Here, we report a pediatric case of Lupus-associated APS in a child who presented as a Cutaneous MVOS. The importance of the current report lies in the awareness of the simultaneous presentation of cutaneous microthrombi in pediatric APS.

## Case presentation

A 7-year-old Iranian boy with no remarkable past medical history was brought to the emergency department with severe pain in his right thumb, agitation, skin color changes, and swelling of his both hands, feet, and lips. The patients and his parents did not note the personal and family history of any rheumatologic disorders or occurrence of thromboembolism accidents.

**Fig. 1:**
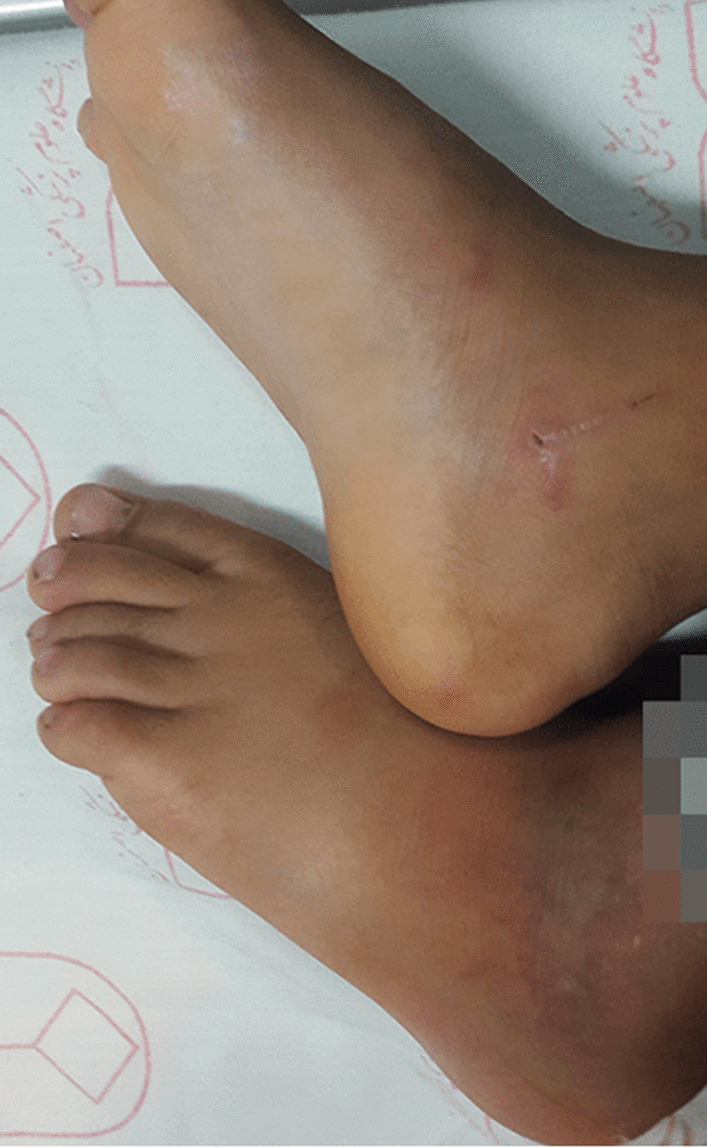
Cutaneous Manifestations. Notable findings included tender, non-blanching, purpuric, and ecchymotic plaques on the medial surface of the right foot

**Fig. 2:**
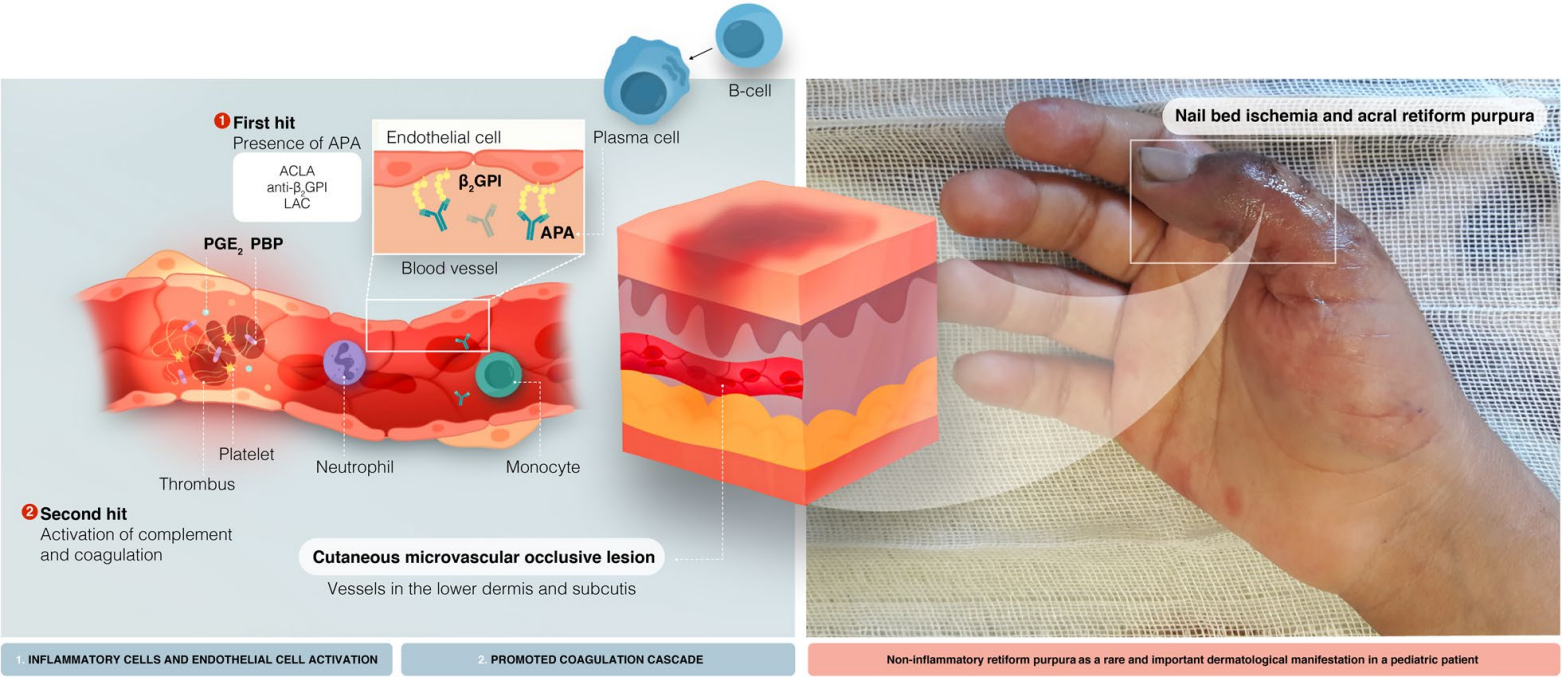
Thrombotic mechanisms mediated by aPL. Vascular dysfunction, inflammatory cell infiltration, and complement deposition all play a critical role in the pathogenesis of APS, as depicted in the illustration. First, the presence of aPL initiates clotting, which in turn activates another procoagulant state, the so-called “second hit”, to induce complete clot formation, which appears to necessitate complement activation in vivo. When immunogenic phospholipid-binding proteins such as β_2_GPI and prothrombin are cross-linked by aPL, cellular activation can be evoked. aPL increases endothelial leukocyte adhesion, cytokine secretion, and PGE_2_ synthesis by upregulating tissue factor expression on endothelial cells and monocytes. Excessive endosomal reactive oxygen species generation in monocytes, the neutrophil release of prothrombotic extracellular traps (NETosis), and complement activation on the surface of endothelium and other cell types significantly intensify inflammation and thrombosis formation. aPL, antiphospholipid autoantibodies; β_2_GPI, β_2_ glycoprotein I; LACA, Lupus anticoagulant antibody; ACLA, anticardiolipin antibody; PGE_2_, prostaglandin E_2_; PBP, phospholipid-binding protein

A week before admission, he experienced gradual development of acral color changes in addition to the bluish discoloration of his lips and tongue in association with low-grade fever; after initiation of symptoms, he was admitted to the hospital for three days and given intravenous cephazolin. Cutaneous examination revealed demarcated bluish discoloration in acral sites, including the ears, hands, feet, and the mouth and extensor surface of the limbs, in combination with intense pain (Figs. 1 and 2). Notable findings included bilaterally, non-blanching, and retiform purpuric plaques on the first digit of the right hand and second and third digits of the right leg and ischemic nail beds of the first finger which were tender. The most prominent lesions measure 2 cm by 3 cm in size.

He had a blood pressure of 110/80 mm Hg and a heart rate of 130 beats per minute. His respiratory rate was 30 breaths/min, with an oxygen saturation of 98% on room air. The distal pulse of all four extremities dose not reveal any abnormality. He rapidly developed dusky red and vesiculobullous formations during the first 24 h of admission, which advanced to the apparent ischemic lesions. The abdomen was neither distended nor tender; there was no lymphadenopathy or hepatosplenomegaly. The oral and vaginal mucosa appeared normal. Aside from the skin lesions, the child's agitation commenced a few hours before being admitted to the hospital, deteriorating his interactiveness. 

During the hospitalization and subsequent follow-up, analyses in the laboratory indicated elevation in the levels of anti-beta-2 glycoprotein I (total anti-β_2_GPI = 19.1 U/ml [Normal range: < 16]), anticardiolipin (aCL IgG = 15.1 U/ml [Normal range: < 10U/ml]) IgG, Lupus Anticoagulant (LAC = 90 [Normal range: < 40]), erythrocyte sedimentation rate (ESR = 103 mm/hr), positive anti-nuclear antibody (ANA), anti-double-stranded DNA (ds-DNA) antibody, and hypocomplementemia were detected during thrombophilia screening (Table 1). No abnormal findings were found in the patient's chest radiography, brain CT scan, abdominal sonography, echocardiography, and limb doppler sonography.

**Table 1:** Laboratory findings of the patient

Test	Result	Unit	Reference interval
D-Dimer	718	ng/mL	Nl: Up to 500
PT	13.4	Sec	11.0–13.0
INR	1.2	N.A	0.9–1.15
PTT	31	Sec	24.0–36.0
FDP	6.8	μg/L	Up to 5
Factor II	72	%	70–120
Factor VII	56	%	60–170
Factor VIII	67	%	60–150
Factor AT III	92	%	80–120
Factor V Leiden	71	%	70–120
Protein C	85	%	65–145
Protein S	81	%	63.5–167.9
Homocysteine	9.6	μmol/L	5–7
LDH	384	U/L	Adult: < 480
BUN	7.7	mg/dL	5.14–16.8
Creatinine	0.4	mg/dL	0.5–1.20
SGOT (AST)	43	U/L	Nl: < 31.0
SGPT (ALT)	10	U/L	Nl: < 31.0
Electrolytes			
Sodium	135	mmol/L	135–145
Potassium	4.7	mmol/L	3.5–5
Inflammatory markers			
CRP	Neg	mg/L	Nl: 0–10
ESR (1 h.)	103	mm/hr	0–10
WBC	23.3	× 10^3^/μL	6.0–17.0
Differential			
Neutrophil	78	%	20–45
Lymphocyte	14	%	40–65
Hemoglobin	11.3	g/dL	11.5–13.5
Platelets (10^3^/l)	407	× 10^3^/μL	130–550
C_3_	1.77	g/L	0.75–1.35
C_4_	0.27	g/L	0.09–0.36

*PTT* Partial Thromboplastin Time, *PT* Prothrombin Time, *INR* International Normalized Ratio, *FDP* Fibrin Degradation Products, *LDH* Lactate Dehydrogenase, *BUN* Blood Urea Nitrogen, *CRP* C-Reactive Protein, *ESR* Erythrocyte Sedimentation Rate

During the first day of admission, there was an alteration in the level of consciousness manifested by agitation, which cannot be explained solely by the discomfort in the purpuric areas of the acral regions.

The patient's treatment plan was supplemented with broad-spectrum antibiotics and three corticosteroid pulses (methylprednisolone 10 mg/kg), which were then converted to oral prednisolone (1 mg/kg/d) on the seventh day of hospitalization, therapeutic heparin. Furthermore, every other day, he received plasmapheresis five times.

There was an improvement in consciousness after 24 h of initiation of the treatment. Ecchymotic lesions resolved on the feet after the sixth day of therapy. Perioral ecchymotic lesions were also alleviated. Based on clinical examination and laboratory findings, the possible diagnosis of cutaneous MVOS is probably in the APS setting (Fig. 2).

**Fig. 3:**
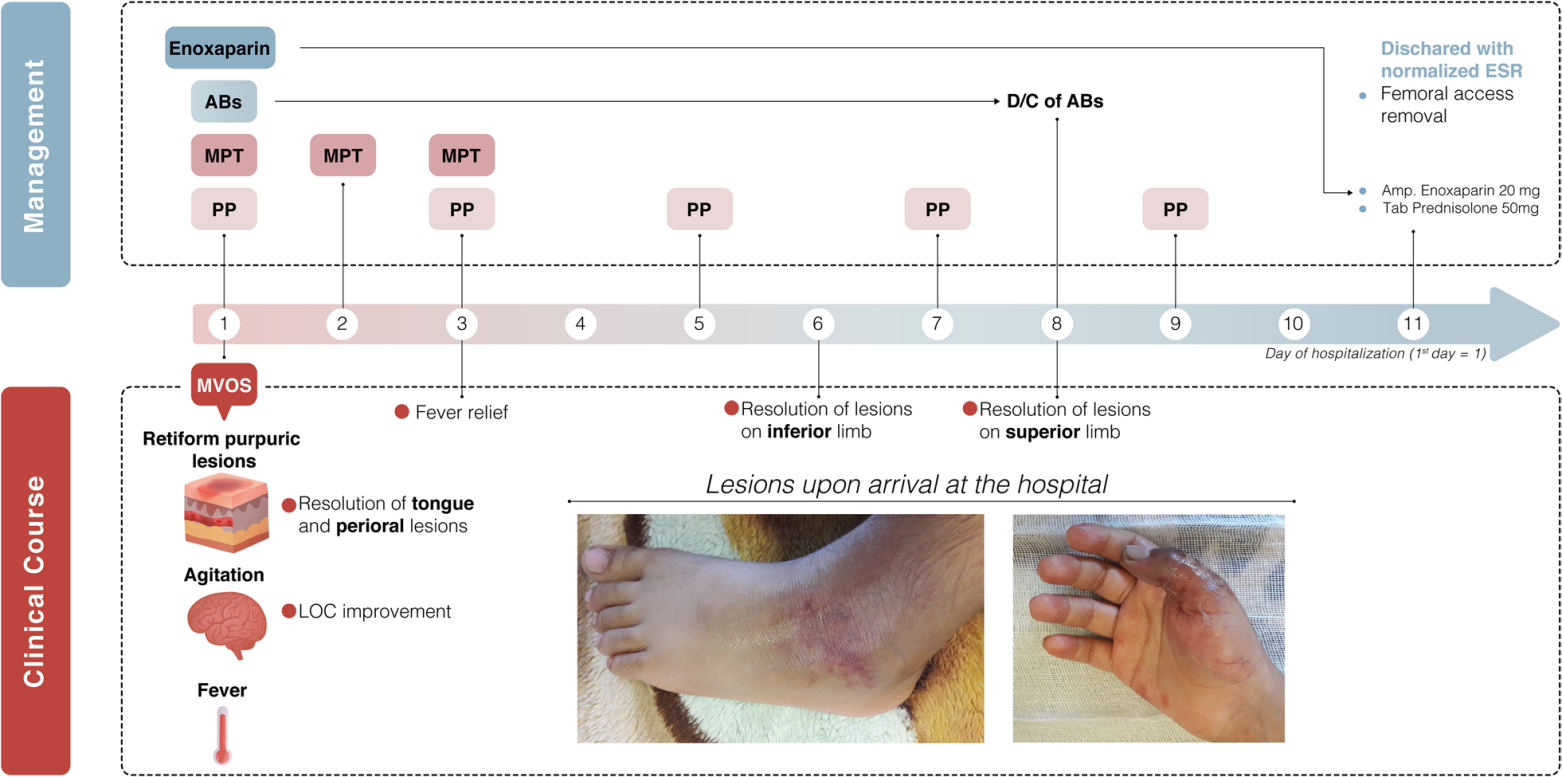
Therapeutic management and clinical course timeline

After 2 weeks of admission, the patient was discharged with prednisolone and enoxaparin and was advised to return to the pediatric rheumatologist. On the follow-up examination and repeat of the laboratory test that showed anti-β2GPI IgG was 1.22, β2GPI IgM was 0.97, and aCL IgG was 2.64, the diagnosis of Lupus-associated APS was made, and the patient underwent regular follow-up (Fig. 3).

## Discussion

We described a child successfully treated with plasmapheresis via femoral access for cutaneous (MVOS) manifested as neurologic involvement, retiform purpura, and digital gangrene subsequent to SLE-associated APS.

Cutaneous MVOS is typically caused by thrombotic, infectious, or embolic phenomena and can be used to identify associated critical systemic conditions and prevent potentially life-threatening and organ-threatening complications through prompt identification and treatment of the comorbid conditions [8]. Cutaneous lesions linked with MVOS are often incorrectly identified as cutaneous vasculitis or misinterpreted as systemic diseases [9]. In CAPS, identification of these clinical manifestations that often precede the occurrence of thrombosis is critical for as soon as possible proper treatment [10]. 

Our patient’s agitation was not exclusively due to the discomfort in the purpuric portions of the acral regions but rather a change in the patient's degree of consciousness. Antiphospholipid antibodies and activated inflammatory mediators may have a role in the etiology of certain neurological correlations, according to studies. APS induces a hypercoagulable condition, which may explain why thromboembolic cerebrovascular episodes are more common in people with APS. Cerebral thrombosis is often the cause of neurological symptoms; thus, many different factors, including elevated levels of aPLs, homocysteine, fibrinogen, protein C, protein S, and antithrombin III, can be considered risk factors [1].

Our patient’s therapeutic approach included a combination of anticoagulation and steroids, as well as attempts to achieve a rapid reduction in aPL titer using plasmapheresis. The infection has been implicated as a contributing triggering factor in CAPS. Fever and leukocytosis at presentation may indicate a condition that precipitated CAPS [2]. As a result, broad-spectrum antibiotics were prescribed for our case and were discontinued after eight days based on negative blood culture results.

Dermatologic characteristics observed in our patient narrow the differential diagnosis to Cutaneous MVOS, which is defined by a clinically significant skin lesion manifesting as retiform purpura and bland necrosis without inflammation. Although dermatologic manifestations are nonspecific and not included in the classification criteria, these abnormalities may be the initial presenting feature, making them essential in identifying this disease [11]. 

APS and cutaneous lesions associated with MVOS are frequently misdiagnosed as cutaneous vasculitis or interpreted as systemic disorders; accordingly, prompt and proper management is critical to the outcome of MVOS [12]. For that reason, it is necessary to be familiar with these cutaneous features and recognize when an APS investigation should be pursued.

## Conclusion

Even though pediatric APS has the potential to cause significant morbidity, it remains a challenging condition. Pediatric APS, probably more frequently than adult APS, is associated with a variety of neurologic, dermatologic, and hematologic symptoms (—both in children with lupus and in children with primary APS. Concludingly, it is essential for pediatricians to be aware of the rare appearance of CAPS as an initial indication of APS. The detailed clinical course, precisely the cutaneous manifestations, should be gathered and studied more thoroughly.

## Data Availability

Upon written request, the corresponding author will provide the data used to support the findings and conclusions.

## Abbreviations

APS: Antiphospholipid syndrome
aPL: antiphospholipid autoantibodies
CAPS: Catastrophic APS
CRP: C-reactive protein
ESR: Erythrocyte sedimentation rate
LAC: Lupus anticoagulant
N.A.: Not applicable
P.T.: Prothrombin time
PTT: Partial thromboplastin time
WBC: White blood cell
AT III: Anti-thrombin III
FDP: Fibrin degradation products
A.B: Antibiotics
MPT: Methylprednisolone pulse therapy
P.P: Plasmapheresis
LOC: Level of consciousness
MVOS: Microvascular occlusion syndrome
